# Poly[[μ_2_-1,2-bis­(4-pyrid­yl)ethene](μ_3_-1,3-phenyl­enediacetato)­cadmium]

**DOI:** 10.1107/S1600536811043467

**Published:** 2011-10-29

**Authors:** Dong Liu

**Affiliations:** a College of Chemistry and Materials Science, Huaibei Normal University, Huaibei 235000, Anhui, People’s Republic of China

## Abstract

In the title coordination polymer, [Cd(C_10_H_8_O_4_)(C_12_H_10_N_2_)]_*n*_, two centrosymmetrically related Cd^II^ atoms are bridged by two 1,3-phenyl­enediacetate ligands forming a chain along the [100] direction. The distorted penta­gonal–bipyramidal coordination about each metal atom is completed by the N atoms of bridging 1,2-bis­(4-pyrid­yl)ethene ligands, which link these one-dimensional chains into a two-dimensional net extending along the (101) plane.

## Related literature

For two-dimensional nets constructed by Cd^II^, dipyridyl ligands and dicarboxyl­ate ligands, see: Tao *et al.* (2003 ▶); Tian *et al.* (2006 ▶); Wang *et al.* (2009 ▶).
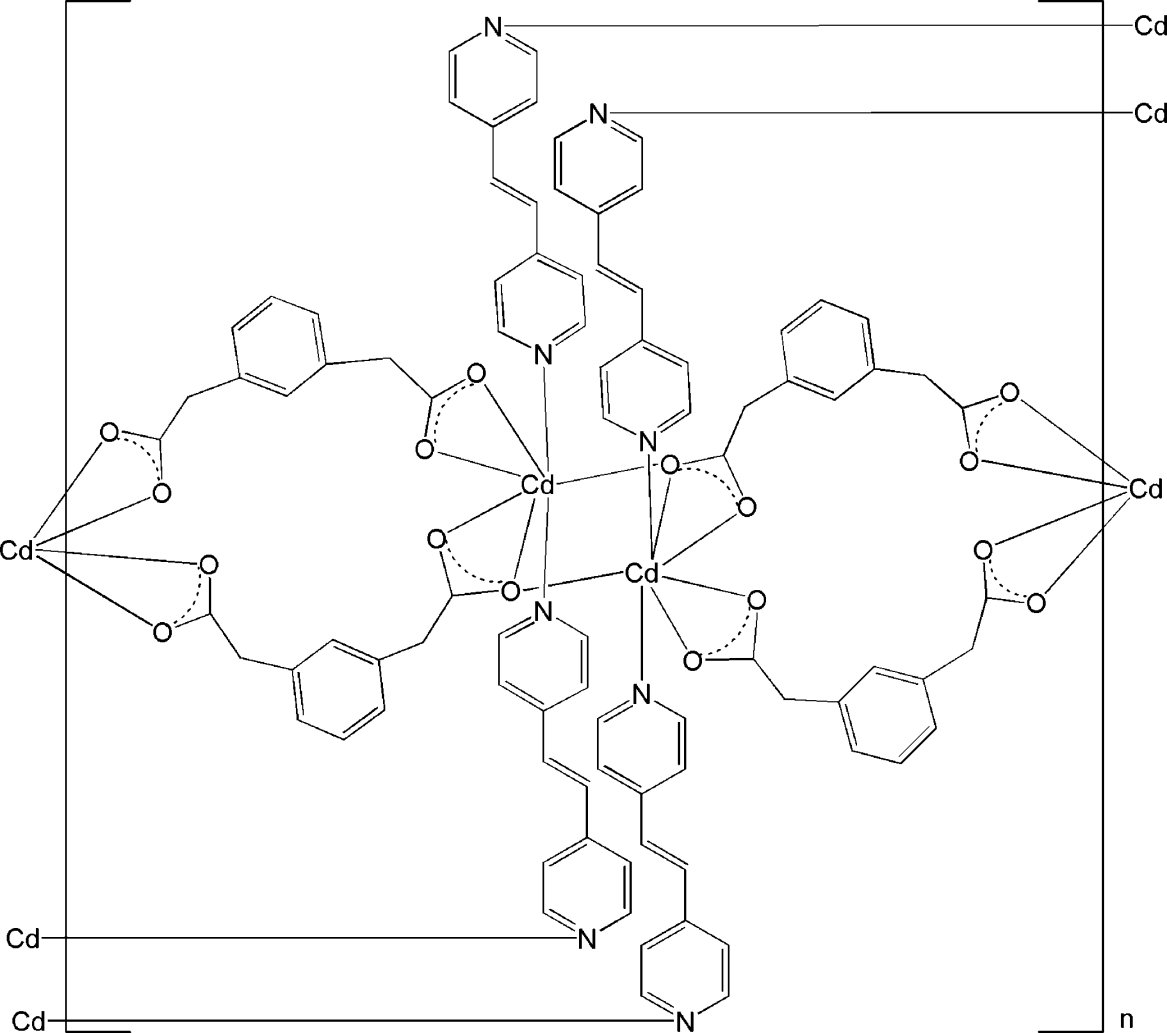


## Experimental

### 

#### Crystal data

[Cd(C_10_H_8_O_4_)(C_12_H_10_N_2_)]
*M* *_r_* = 486.79
Triclinic, 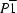

*a* = 9.4626 (19) Å
*b* = 10.113 (2) Å
*c* = 11.351 (2) Å
α = 98.95 (3)°
β = 92.19 (3)°
γ = 116.88 (3)°
*V* = 949.8 (3) Å^3^
*Z* = 2
Mo *K*α radiation
μ = 1.18 mm^−1^
*T* = 223 K
0.40 × 0.30 × 0.25 mm

#### Data collection

Rigaku MercuryCCD area-detector diffractometer
Absorption correction: multi-scan (*REQAB*; Jacobson, 1998 ▶) *T*_min_ = 0.649, *T*_max_ = 0.757
8582 measured reflections
4256 independent reflections
3630 reflections with *I* > 2σ(*I*)
*R*_int_ = 0.029

#### Refinement

*R*[*F* ^2^ > 2σ(*F* ^2^)] = 0.033
*wR*(*F* ^2^) = 0.083
*S* = 1.09
4256 reflections
263 parameters
H-atom parameters constrained
Δρ_max_ = 1.16 e Å^−3^
Δρ_min_ = −0.85 e Å^−3^

### 

Data collection: *CrystalClear* (Rigaku, 2001 ▶); cell refinement: *CrystalClear*; data reduction: *CrystalStructure* (Rigaku/MSC, 2004 ▶); program(s) used to solve structure: *SHELXTL* (Sheldrick, 2008 ▶); program(s) used to refine structure: *SHELXTL*; molecular graphics: *SHELXTL*; software used to prepare material for publication: *SHELXTL* and *PLATON* (Spek, 2009 ▶).

## Supplementary Material

Crystal structure: contains datablock(s) I, global. DOI: 10.1107/S1600536811043467/bq2313sup1.cif

Structure factors: contains datablock(s) I. DOI: 10.1107/S1600536811043467/bq2313Isup2.hkl

Additional supplementary materials:  crystallographic information; 3D view; checkCIF report

## Figures and Tables

**Fig. 1.**
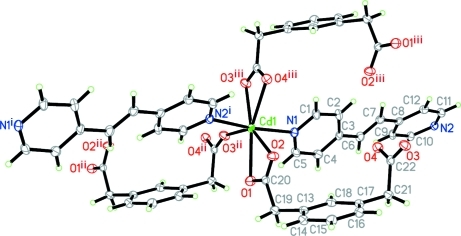
Coordination environment of CdII atom in the compound with nonhydrogen atoms represented by thermal ellipsoids draw at 30% probability level. [Symmetry codes: i: x, y + 1, z + 1; ii: x – 1, y, z; iii: – x + 1, – y + 1, – z.]

**Fig. 2.**
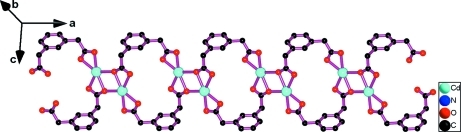
View of the one-dimensional chain in the title compound.

**Fig. 3.**
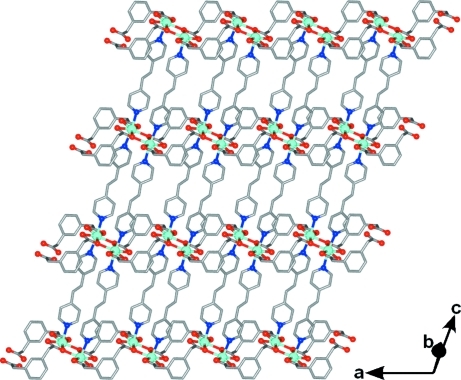
View of the two-dimensional net of the title compound.

## supplementary crystallographic information

### Comment

In recent years, particular attention has been devoted to coordination polymers because of their undisputed beauty and potential applications as materials for adsorption, separation, and catalysis (Tao *et al.*, 2003; Tian *et al.*, 2006; Wang *et al.*, 2009). Conformationally flexible dicarboxylate ligands, showing varied geometries, are often featured in these new classes of compounds (Wang *et al.*, 2009).

In this work, the reaction between Cd(NO_3_)_2_, 1,3-phenylenediacetic acid (1,3-H_2_pda) and 1,2-bis(4-pyridyl)ethene (bpe) afforded the title coordination polymer, [Cd(C_12_H_10_N_2_)(C_10_H_8_O_4_)]_n_ (I). In (I), each Cd^II^ atom is located in a pentagonal bipyramidally environment, coordinated by five O atoms from three different 1,3-pda ligands at the basal positions and two N atom from two different bpe ligands at the apical position (Fig. 1). Two centrosymmetrically related Cd^II^ atoms are linked by two 1,3-pda ligands to form a one-dimensional chain along the *a* axis (Fig. 2). Such a chain is connected to its adjacent ones *via* pairs of bpe ligands to form a two-dimensional net extending along the *ac* plane (Fig. 3).

### Experimental

To a 25 ml Teflon-lined stainless steel autoclave was loaded Cd(NO_3_)_2_.4H_2_O (154 mg, 0.5 mmol), 1,3-phenylenediacetic acid (97 mg, 0.5 mmol), 1,2-bis(4-pyridyl)ethene (91 mg, 0.5 mmol), NaOH (40 mg, 1 mmol) and H_2_O (15 ml). The autoclave was sealed and heated in an oven to 433 K for three days, and then cooled to ambient temperature at the rate of 5 K/h to form yellow crystals. Yield: 180 mg (74% yield based on Cd). Anal. calcd. for C_22_H_18_CdN_2_O_4_: C, 54.28; H, 3.73; N, 5.75. Found: C, 53.96; H, 3.77; N, 6.03.

### Refinement

The C-bound H atoms were positioned geometrically, with C–H = 0.97 Å (methylene) or 0.94 Å (phenyl, pyridyl and vinyl), and refined as riding, with *U*_iso_(H) = 1.5*U*_eq_(C) for methylene groups or 1.2*U*_eq_(C) otherwise.

### Crystal data

**Table:**

[Cd(C_10_H_8_O_4_)(C_12_H_10_N_2_)]	*Z* = 2
*M**_r_* = 486.79	*F*(000) = 488
Triclinic, *P*1	*D*_x_ = 1.702 Mg m^−^^3^
Hall symbol: -P 1	Mo *K*α radiation, λ = 0.71073 Å
*a* = 9.4626 (19) Å	Cell parameters from 4814 reflections
*b* = 10.113 (2) Å	θ = 3.2–27.5°
*c* = 11.351 (2) Å	µ = 1.18 mm^−^^1^
α = 98.95 (3)°	*T* = 223 K
β = 92.19 (3)°	Block, yellow
γ = 116.88 (3)°	0.40 × 0.30 × 0.25 mm
*V* = 949.8 (3) Å^3^	

### Data collection

**Table:**

Rigaku MercuryCCD area-detector diffractometer	4256 independent reflections
Radiation source: fine-focus sealed tube	3630 reflections with *I* > 2σ(*I*)
graphite	*R*_int_ = 0.029
ω scans	θ_max_ = 27.5°, θ_min_ = 3.2°
Absorption correction: multi-scan (REQAB; Jacobson, 1998)	*h* = −12→11
*T*_min_ = 0.649, *T*_max_ = 0.757	*k* = −12→12
8582 measured reflections	*l* = −11→14

### Refinement

**Table:**

Refinement on *F*^2^	Secondary atom site location: difference Fourier map
Least-squares matrix: full	Hydrogen site location: inferred from neighbouring sites
*R*[*F*^2^ > 2σ(*F*^2^)] = 0.033	H-atom parameters constrained
*wR*(*F*^2^) = 0.083	*w* = 1/[σ^2^(*F*_o_^2^) + (0.0352*P*)^2^] where *P* = (*F*_o_^2^ + 2*F*_c_^2^)/3
*S* = 1.09	(Δ/σ)_max_ = 0.001
4256 reflections	Δρ_max_ = 1.16 e Å^−^^3^
263 parameters	Δρ_min_ = −0.85 e Å^−^^3^
0 restraints	Extinction correction: *SHELXTL* (Sheldrick, 2008), Fc^*^=kFc[1+0.001xFc^2^λ^3^/sin(2θ)]^-1/4^
Primary atom site location: structure-invariant direct methods	Extinction coefficient: 0.098 (3)

### Special details

**Table:**

Geometry. All e.s.d.'s (except the e.s.d. in the dihedral angle between two l.s. planes) are estimated using the full covariance matrix. The cell e.s.d.'s are taken into account individually in the estimation of e.s.d.'s in distances, angles and torsion angles; correlations between e.s.d.'s in cell parameters are only used when they are defined by crystal symmetry. An approximate (isotropic) treatment of cell e.s.d.'s is used for estimating e.s.d.'s involving l.s. planes.
Refinement. Refinement of *F*^2^ against ALL reflections. The weighted *R*-factor *wR* and goodness of fit *S* are based on *F*^2^, conventional *R*-factors *R* are based on *F*, with *F* set to zero for negative *F*^2^. The threshold expression of *F*^2^ > σ(*F*^2^) is used only for calculating *R*-factors(gt) *etc*. and is not relevant to the choice of reflections for refinement. *R*-factors based on *F*^2^ are statistically about twice as large as those based on *F*, and *R*- factors based on ALL data will be even larger.

### Fractional atomic coordinates and isotropic or equivalent isotropic displacement parameters (Å^2^)

**Table:**

	*x*	*y*	*z*	*U*_iso_*/*U*_eq_	
Cd1	0.16456 (3)	0.49799 (2)	0.10431 (2)	0.02493 (12)	
N1	0.1884 (3)	0.3281 (3)	−0.0471 (3)	0.0287 (6)	
N2	0.1751 (3)	−0.3077 (3)	−0.7491 (2)	0.0266 (6)	
O1	0.1024 (3)	0.3142 (3)	0.2322 (2)	0.0353 (6)	
O2	0.3550 (3)	0.4760 (2)	0.2348 (2)	0.0350 (6)	
O3	0.8868 (3)	0.3906 (3)	0.0535 (2)	0.0362 (6)	
O4	0.6363 (3)	0.2894 (3)	−0.0233 (2)	0.0336 (6)	
C1	0.2978 (4)	0.3687 (4)	−0.1239 (3)	0.0338 (8)	
H1	0.3788	0.4693	−0.1097	0.041*	
C2	0.2974 (4)	0.2702 (4)	−0.2224 (3)	0.0325 (8)	
H2	0.3759	0.3039	−0.2745	0.039*	
C3	0.1791 (4)	0.1192 (3)	−0.2445 (3)	0.0288 (7)	
C4	0.0700 (4)	0.0771 (4)	−0.1623 (3)	0.0355 (8)	
H4	−0.0090	−0.0239	−0.1714	0.043*	
C5	0.0775 (5)	0.1841 (4)	−0.0670 (3)	0.0365 (9)	
H5	0.0006	0.1536	−0.0135	0.044*	
C6	0.1591 (4)	0.0064 (4)	−0.3510 (3)	0.0337 (8)	
H6	0.0776	−0.0924	−0.3543	0.040*	
C7	0.2438 (4)	0.0293 (4)	−0.4427 (3)	0.0320 (8)	
H7	0.3282	0.1267	−0.4391	0.038*	
C8	0.2160 (4)	−0.0861 (3)	−0.5501 (3)	0.0291 (7)	
C9	0.3390 (4)	−0.0707 (4)	−0.6182 (3)	0.0315 (8)	
H9	0.4385	0.0162	−0.5989	0.038*	
C10	0.3148 (4)	−0.1832 (4)	−0.7145 (3)	0.0315 (8)	
H10	0.4008	−0.1718	−0.7580	0.038*	
C11	0.0541 (4)	−0.3206 (4)	−0.6863 (3)	0.0299 (7)	
H11	−0.0458	−0.4061	−0.7103	0.036*	
C12	0.0698 (4)	−0.2139 (4)	−0.5878 (3)	0.0331 (8)	
H12	−0.0183	−0.2276	−0.5462	0.040*	
C13	0.4592 (4)	0.2971 (3)	0.3492 (3)	0.0284 (7)	
C14	0.5692 (4)	0.3628 (4)	0.4528 (3)	0.0323 (8)	
H14	0.5405	0.4004	0.5239	0.039*	
C15	0.7199 (4)	0.3733 (4)	0.4519 (3)	0.0358 (8)	
H15	0.7939	0.4199	0.5218	0.043*	
C16	0.7619 (4)	0.3156 (4)	0.3491 (3)	0.0347 (8)	
H16	0.8649	0.3239	0.3490	0.042*	
C17	0.6528 (4)	0.2449 (4)	0.2447 (3)	0.0301 (8)	
C18	0.5032 (4)	0.2396 (3)	0.2462 (3)	0.0305 (8)	
H18	0.4304	0.1959	0.1757	0.037*	
C19	0.2923 (4)	0.2811 (4)	0.3494 (3)	0.0320 (8)	
H19A	0.2798	0.3177	0.4316	0.038*	
H19B	0.2160	0.1733	0.3279	0.038*	
C20	0.2475 (4)	0.3636 (3)	0.2656 (3)	0.0243 (7)	
C21	0.6985 (4)	0.1814 (4)	0.1321 (3)	0.0334 (8)	
H21A	0.6091	0.0842	0.0934	0.040*	
H21B	0.7899	0.1645	0.1525	0.040*	
C22	0.7419 (4)	0.2925 (3)	0.0462 (3)	0.0265 (7)	

### Atomic displacement parameters (Å^2^)

**Table:**

	*U*^11^	*U*^22^	*U*^33^	*U*^12^	*U*^13^	*U*^23^
Cd1	0.03223 (17)	0.02483 (16)	0.01910 (16)	0.01568 (11)	0.00324 (10)	0.00013 (9)
N1	0.0356 (15)	0.0267 (14)	0.0227 (16)	0.0157 (12)	0.0049 (13)	−0.0021 (11)
N2	0.0348 (15)	0.0267 (13)	0.0190 (15)	0.0167 (12)	0.0024 (12)	−0.0013 (11)
O1	0.0246 (12)	0.0387 (13)	0.0438 (17)	0.0143 (10)	0.0011 (11)	0.0135 (11)
O2	0.0269 (12)	0.0334 (12)	0.0422 (16)	0.0094 (10)	0.0020 (11)	0.0162 (11)
O3	0.0282 (13)	0.0411 (14)	0.0372 (16)	0.0120 (11)	0.0061 (11)	0.0143 (12)
O4	0.0324 (13)	0.0397 (13)	0.0278 (15)	0.0161 (11)	0.0016 (11)	0.0067 (11)
C1	0.0317 (18)	0.0310 (17)	0.033 (2)	0.0129 (15)	0.0063 (16)	−0.0055 (15)
C2	0.0274 (17)	0.0378 (18)	0.028 (2)	0.0147 (15)	0.0067 (15)	−0.0035 (15)
C3	0.0362 (18)	0.0281 (16)	0.026 (2)	0.0199 (15)	0.0030 (15)	−0.0008 (14)
C4	0.047 (2)	0.0230 (16)	0.030 (2)	0.0120 (15)	0.0084 (17)	0.0004 (14)
C5	0.049 (2)	0.0311 (18)	0.027 (2)	0.0164 (16)	0.0148 (17)	0.0029 (15)
C6	0.039 (2)	0.0275 (17)	0.032 (2)	0.0159 (15)	0.0034 (16)	−0.0022 (14)
C7	0.041 (2)	0.0253 (16)	0.029 (2)	0.0159 (15)	0.0035 (16)	0.0020 (14)
C8	0.043 (2)	0.0264 (16)	0.0231 (19)	0.0208 (15)	0.0025 (15)	0.0031 (14)
C9	0.042 (2)	0.0247 (16)	0.024 (2)	0.0134 (15)	0.0034 (16)	0.0008 (14)
C10	0.0374 (19)	0.0318 (17)	0.0236 (19)	0.0159 (15)	0.0063 (15)	0.0007 (14)
C11	0.0329 (18)	0.0315 (17)	0.0247 (19)	0.0164 (15)	0.0010 (15)	−0.0008 (14)
C12	0.0355 (19)	0.0428 (19)	0.027 (2)	0.0245 (16)	0.0076 (16)	0.0031 (15)
C13	0.0299 (17)	0.0306 (17)	0.028 (2)	0.0145 (14)	0.0065 (15)	0.0121 (15)
C14	0.0363 (19)	0.0346 (18)	0.028 (2)	0.0177 (15)	0.0081 (16)	0.0082 (15)
C15	0.038 (2)	0.0413 (19)	0.030 (2)	0.0211 (17)	−0.0032 (16)	0.0042 (16)
C16	0.0314 (18)	0.0425 (19)	0.039 (2)	0.0221 (16)	0.0070 (17)	0.0140 (17)
C17	0.0367 (19)	0.0299 (17)	0.030 (2)	0.0175 (15)	0.0113 (16)	0.0138 (15)
C18	0.0331 (18)	0.0315 (17)	0.026 (2)	0.0135 (15)	0.0033 (15)	0.0097 (15)
C19	0.0284 (17)	0.0367 (18)	0.031 (2)	0.0137 (15)	0.0060 (15)	0.0119 (15)
C20	0.0279 (17)	0.0261 (15)	0.0211 (18)	0.0148 (14)	0.0042 (14)	0.0035 (13)
C21	0.041 (2)	0.0321 (17)	0.031 (2)	0.0188 (16)	0.0109 (17)	0.0094 (15)
C22	0.0338 (18)	0.0285 (16)	0.0193 (18)	0.0177 (15)	0.0052 (15)	−0.0010 (13)

### Geometric parameters (Å, °)

**Table:**

Cd1—N1	2.327 (3)	C6—H6	0.9400
Cd1—N2^i^	2.332 (3)	C7—C8	1.474 (5)
Cd1—O3^ii^	2.351 (2)	C7—H7	0.9400
Cd1—O2	2.398 (2)	C8—C9	1.386 (5)
Cd1—O3^iii^	2.402 (2)	C8—C12	1.390 (5)
Cd1—O1	2.413 (2)	C9—C10	1.378 (5)
Cd1—O4^iii^	2.470 (2)	C9—H9	0.9400
Cd1—C20	2.729 (3)	C10—H10	0.9400
Cd1—C22^iii^	2.781 (3)	C11—C12	1.380 (5)
N1—C5	1.331 (4)	C11—H11	0.9400
N1—C1	1.340 (4)	C12—H12	0.9400
N2—C10	1.337 (4)	C13—C18	1.387 (5)
N2—C11	1.338 (4)	C13—C14	1.395 (5)
N2—Cd1^iv^	2.331 (3)	C13—C19	1.513 (5)
O1—C20	1.249 (4)	C14—C15	1.382 (5)
O2—C20	1.247 (4)	C14—H14	0.9400
O3—C22	1.267 (4)	C15—C16	1.376 (5)
O3—Cd1^v^	2.351 (2)	C15—H15	0.9400
O3—Cd1^iii^	2.402 (2)	C16—C17	1.399 (5)
O4—C22	1.235 (4)	C16—H16	0.9400
O4—Cd1^iii^	2.470 (2)	C17—C18	1.392 (5)
C1—C2	1.376 (5)	C17—C21	1.507 (5)
C1—H1	0.9400	C18—H18	0.9400
C2—C3	1.400 (4)	C19—C20	1.522 (5)
C2—H2	0.9400	C19—H19A	0.9800
C3—C4	1.385 (5)	C19—H19B	0.9800
C3—C6	1.469 (5)	C21—C22	1.529 (5)
C4—C5	1.380 (5)	C21—H21A	0.9800
C4—H4	0.9400	C21—H21B	0.9800
C5—H5	0.9400	C22—Cd1^iii^	2.781 (3)
C6—C7	1.323 (5)		
			
N1—Cd1—N2^i^	172.12 (9)	C3—C4—H4	120.1
N1—Cd1—O3^ii^	92.92 (10)	N1—C5—C4	123.3 (3)
N2^i^—Cd1—O3^ii^	93.60 (10)	N1—C5—H5	118.4
N1—Cd1—O2	88.91 (10)	C4—C5—H5	118.4
N2^i^—Cd1—O2	88.96 (10)	C7—C6—C3	126.8 (3)
O3^ii^—Cd1—O2	139.86 (8)	C7—C6—H6	116.6
N1—Cd1—O3^iii^	86.43 (9)	C3—C6—H6	116.6
N2^i^—Cd1—O3^iii^	91.45 (9)	C6—C7—C8	125.0 (3)
O3^ii^—Cd1—O3^iii^	71.46 (9)	C6—C7—H7	117.5
O2—Cd1—O3^iii^	148.59 (8)	C8—C7—H7	117.5
N1—Cd1—O1	88.59 (9)	C9—C8—C12	116.8 (3)
N2^i^—Cd1—O1	96.33 (9)	C9—C8—C7	120.0 (3)
O3^ii^—Cd1—O1	85.54 (8)	C12—C8—C7	123.1 (3)
O2—Cd1—O1	54.39 (8)	C10—C9—C8	119.7 (3)
O3^iii^—Cd1—O1	156.16 (8)	C10—C9—H9	120.1
N1—Cd1—O4^iii^	89.83 (9)	C8—C9—H9	120.1
N2^i^—Cd1—O4^iii^	82.82 (9)	N2—C10—C9	123.3 (3)
O3^ii^—Cd1—O4^iii^	124.49 (8)	N2—C10—H10	118.4
O2—Cd1—O4^iii^	95.59 (8)	C9—C10—H10	118.4
O3^iii^—Cd1—O4^iii^	53.40 (8)	N2—C11—C12	122.8 (3)
O1—Cd1—O4^iii^	149.96 (8)	N2—C11—H11	118.6
N1—Cd1—C20	87.53 (10)	C12—C11—H11	118.6
N2^i^—Cd1—C20	94.02 (10)	C11—C12—C8	120.0 (3)
O3^ii^—Cd1—C20	112.79 (9)	C11—C12—H12	120.0
O2—Cd1—C20	27.18 (8)	C8—C12—H12	120.0
O3^iii^—Cd1—C20	172.80 (8)	C18—C13—C14	118.6 (3)
O1—Cd1—C20	27.25 (8)	C18—C13—C19	120.1 (3)
O4^iii^—Cd1—C20	122.72 (9)	C14—C13—C19	121.3 (3)
N1—Cd1—C22^iii^	88.67 (9)	C15—C14—C13	120.6 (3)
N2^i^—Cd1—C22^iii^	86.02 (9)	C15—C14—H14	119.7
O3^ii^—Cd1—C22^iii^	98.26 (10)	C13—C14—H14	119.7
O2—Cd1—C22^iii^	121.88 (9)	C16—C15—C14	120.2 (4)
O3^iii^—Cd1—C22^iii^	27.06 (9)	C16—C15—H15	119.9
O1—Cd1—C22^iii^	175.42 (8)	C14—C15—H15	119.9
O4^iii^—Cd1—C22^iii^	26.36 (9)	C15—C16—C17	120.6 (3)
C20—Cd1—C22^iii^	148.87 (10)	C15—C16—H16	119.7
N1—Cd1—Cd1^vi^	89.56 (8)	C17—C16—H16	119.7
N2^i^—Cd1—Cd1^vi^	93.09 (8)	C18—C17—C16	118.3 (3)
O3^ii^—Cd1—Cd1^vi^	36.17 (6)	C18—C17—C21	120.9 (3)
O2—Cd1—Cd1^vi^	175.62 (5)	C16—C17—C21	120.7 (3)
O3^iii^—Cd1—Cd1^vi^	35.29 (6)	C13—C18—C17	121.6 (3)
O1—Cd1—Cd1^vi^	121.47 (6)	C13—C18—H18	119.2
O4^iii^—Cd1—Cd1^vi^	88.51 (6)	C17—C18—H18	119.2
C20—Cd1—Cd1^vi^	148.61 (7)	C13—C19—C20	116.1 (3)
C22^iii^—Cd1—Cd1^vi^	62.17 (8)	C13—C19—H19A	108.3
C5—N1—C1	117.3 (3)	C20—C19—H19A	108.3
C5—N1—Cd1	118.4 (2)	C13—C19—H19B	108.3
C1—N1—Cd1	124.0 (2)	C20—C19—H19B	108.3
C10—N2—C11	117.2 (3)	H19A—C19—H19B	107.4
C10—N2—Cd1^iv^	118.7 (2)	O2—C20—O1	123.5 (3)
C11—N2—Cd1^iv^	123.5 (2)	O2—C20—C19	119.4 (3)
C20—O1—Cd1	90.57 (19)	O1—C20—C19	117.1 (3)
C20—O2—Cd1	91.3 (2)	O2—C20—Cd1	61.47 (17)
C22—O3—Cd1^v^	156.6 (2)	O1—C20—Cd1	62.18 (17)
C22—O3—Cd1^iii^	93.4 (2)	C19—C20—Cd1	176.6 (2)
Cd1^v^—O3—Cd1^iii^	108.54 (9)	C17—C21—C22	109.4 (3)
C22—O4—Cd1^iii^	91.02 (19)	C17—C21—H21A	109.8
N1—C1—C2	123.2 (3)	C22—C21—H21A	109.8
N1—C1—H1	118.4	C17—C21—H21B	109.8
C2—C1—H1	118.4	C22—C21—H21B	109.8
C1—C2—C3	119.4 (3)	H21A—C21—H21B	108.2
C1—C2—H2	120.3	O4—C22—O3	122.1 (3)
C3—C2—H2	120.3	O4—C22—C21	120.0 (3)
C4—C3—C2	116.9 (3)	O3—C22—C21	117.7 (3)
C4—C3—C6	118.5 (3)	O4—C22—Cd1^iii^	62.63 (17)
C2—C3—C6	124.5 (3)	O3—C22—Cd1^iii^	59.56 (17)
C5—C4—C3	119.8 (3)	C21—C22—Cd1^iii^	177.3 (3)
C5—C4—H4	120.1		

Symmetry codes: (i) *x*, *y*+1, *z*+1; (ii) *x*−1, *y*, *z*; (iii) −*x*+1, −*y*+1, −*z*; (iv) *x*, *y*−1, *z*−1; (v) *x*+1, *y*, *z*; (vi) −*x*, −*y*+1, −*z*.
